# Multiparametric MRI model with synthetic MRI, DWI multi-quantitative parameters, and differential sub-sampling with cartesian ordering enables BI-RADS 4 lesions diagnosis with high accuracy

**DOI:** 10.3389/fonc.2023.1180131

**Published:** 2024-01-05

**Authors:** Hua He, Meina Song, Zhaorong Tian, Na Gao, Jiale Ma, Zhijun Wang

**Affiliations:** ^1^ Department of Radiology, General Hospital of First Clinical Medical College, Ningxia Medical University, Yinchuan, China; ^2^ First Clinical Medical College, Ningxia Medical University, Yinchuan, China

**Keywords:** synthetic magnetic resonance imaging (syMRI), magnetic resonance imaging, nomograms, breast cancer, BI-RANDS 4

## Abstract

**Objective:**

To assess the feasibility and diagnostic performances of synthetic magnetic resonance imaging (SyMRI) combined with diffusion-weighted imaging (DWI) and differential subsampling with cartesian ordering (DISCO) in breast imaging reporting and data system (BI-RADS) 4 lesions.

**Methods:**

A total of 98 BI-RADS 4 patients, including 68 cases assigned to a malignant group and 33 cases assigned to a benign group, were prospectively enrolled, and their MRI and clinical information were collected. Two physicians jointly analyzed the characteristics of conventional MRI. T1, T2, proton density (PD), and ADC values were obtained from three different regions of interest (ROIs). Logistic regression analyses were used to select features and build models, and a nomogram was constructed with the best model.

**Results:**

Using the ROI delineation method at the most obvious enhancement to measure the ADC value revealed the best diagnostic performance in diagnosing BI-RADS type 4 mass lesions. The diagnostic efficiency of the maximum level drawing method of the quantitative relaxation model was better than that of the whole drawing method and the most obvious enhancement method. The best relaxation model (model A) was composed of two parameters: T2_stand_ and ΔT1%_stand_ (AUC=0.887), and the BI-RADS model (model B) was constructed by two MRI features of edge and TIC curve (AUC=0.793). Using the quantitative parameters of SyMRI and DWI of the best ROC method combined with DISCO enhanced MRI features to establish a joint diagnostic model (model C: edge, TIC curve type, ADC_local_, T2_stand_, ΔT1%_stand_) showed the best diagnostic efficiency (AUC=0.953). The nomogram also had calibration curves with good overlap

**Conclusions:**

The combined diagnosis model of SyMRI and DWI quantitative parameters combined with DISCO can improve the diagnostic efficiency of BI-RADS 4 types of mass lesions. Also, the line diagram based on this model can be used as an auxiliary diagnostic tool.

## Highlights

• The diagnostic efficiency of the maximum level drawing method of the quantitative relaxation model is better than that of the whole drawing method and the most obvious enhancement method. The maximum level drawing method is simple, repeatable, and suitable for popularization.
• The ADC value can be used to quantitatively evaluate the limited degree of molecular water diffusion in living tissues. Also, using the ROI delineation method at the most obvious enhancement to measure the ADC value has the best diagnostic performance in diagnosing BI-RADS type 4 mass lesions.
• The combined diagnosis model of SyMRI and DWI quantitative parameters combined with DISCO can further improve the diagnostic efficiency of BI-RADS four types of mass lesions, and the nomogram established based on this model can be used as an auxiliary diagnostic tool.

## Introduction

Breast cancer is the most common malignant tumor affecting women worldwide and the main cause of cancer death among women (1, 2). The cure rate of early breast cancer is more than 90%; however, once recurrence and metastasis occur, the survival time of patients is only 18-30 months (3). Early diagnosis and treatment of breast cancer can significantly prolong the survival time of patients and reduce the cost of treatment (4). Accordingly, it is critical to differentiate benign and malignant breast lesions, especially in the early stage, which could be facilitated by developing advanced imaging techniques and efficient auxiliary diagnosis tools.

Breast imaging reporting and data system (BI-RADS) is a standardized guide for breast disease image reporting proposed by the American Society of Radiology (ACR). In the latest 5th edition of the BI-RADS dictionary, BI-RADS class 4 lesions are suspected malignant lesions; however, the range of malignant transformation is wide, and the degree of malignancy is about 2% ~ 94% (5). DCE-MRI has great value in the differential diagnosis of benign and malignant breast lesions, in addition to being the core sequence of BI-RADS. It is mainly evaluated according to the morphology and limited hemodynamic signs of the lesions (6). Although it can help clinical decision-making of breast lesions, the morphological and hemodynamic characteristics of some benign and malignant lesions overlap, and the specificity is low (7). In particular, for BI-RADS class 4 lesions, conventional MRI examination of benign and malignant lesions does not reveal the typical manifestations of breast cancer, so clinical biopsy is often needed to make a definite diagnosis.

DWI is an important supplementary diagnostic sequence for breast lesions whose quantitative index, ADC value, can be used to diagnose the nature of the lesions quantitatively. Nonetheless, it cannot detect information about the composition of the microstructure. Compared with the average specificity of DCE-MRI for breast cancer diagnosis, which is some 80%, the average specificity of the combined diagnosis of DCE-MRI and DWI can reach 89.2% (8). Therefore, the application of new nuclear magnetic resonance technology and multi-quantitative parameters to improve the diagnostic accuracy of BI-RADS class 4 lesions has become very important in clinical work.

Differential subsampling with cartesian ordering (DISCO) is a multi-phase dynamic contrast enhancement imaging technique with high time and spatial resolution. DISCO technology adopts K-space random under acquisition and time-domain multiplexing technology to minimize the mutual restriction between time and spatial resolution and obtain more accurate TIC curves and more accurate enhancement characteristics of lesions (9, 10).

SyMRI is a new MR imaging method that is excited by saturated pulses and produces fast spin echoes. The read multi-phase dynamic multi-echo (MDME) sequence is used for signal acquisition. Five kinds of quantitative maps and various images with different contrasts can be obtained by one scan, thus satisfying both morphological and quantitative tissue evaluation of relaxation time and permitting objective judgment (11–13). Several studies have proved that SyMRI can be used to distinguish benign and malignant breast lesions (14). In their study, Matsuda et al. found that a combination of synthetic MRI and DCE-MRI improves the diagnosis of benign and malignant breast masses, especially category 4 masses (15). However, the measurement methods of quantitative parameters lack unified operating standards, which leads to differences in the measurement results from different observers (15, 16). Furthermore, there are only a few studies on the impact of syMRI measurement methods on the diagnosis of benign and malignant breast lesions. Accordingly, this study aimed to investigate the value of SyMRI and DWI quantitative parameters of the best ROC method combined with DISCO enhancement in the differential diagnosis of BI-RADS class 4 benign and malignant lesions.

## Methods

### Study design and population

Data and pathological results were collected from 98 female patients between July 2020 and December 2021. The prospective study was approved by the Ethics Committee of the General Hospital of Ningxia Medical University, and all the subjects signed an informed consent form.

Inclusion criteria were the following: (1) all patients had mass lesions, which were classified as BI-RADS 4 in the MRI imaging diagnosis report; (2) the preoperative imaging data were complete; (3) they did not receive surgery, radiation, chemical and hormonal treatment before MRI examination. Exclusion criteria were: (1) poor image quality or impossibility to sketch ROI; (2) both benign and malignant lesions in the ipsilateral breast. When there were multiple lesions in one breast of the same patient, the lesion with the largest diameter was included in the analysis.

### Procedures

All MR examinations were performed on a 3.0-T MRI system (SIGNA™ Architect, GE Healthcare) with an eight-channel phased-array breast coil. All patients underwent T1WI, T2WI, DWI (b value is 800s/mm^2^), multi-phase DISCO enhancement, and syMRI before and after enhancement (MAGiC). The scan parameters of MAGiC were the same before and after enhancement. Gd-DTPA-BMA (Omni Scan, GE Healthcare, Ireland) was injected at a dose of 0.1mmol/kg and an injection rate of 2.5ml/s, after which the tube was flushed with the same amount of normal saline, and 15 images were continuously collected without interval. The total scanning time was 8 min 40s.

According to the 5th edition BI-RADS MRI lexicon, two physicians with respectively 10 and 5 years of experience in breast imaging diagnosis reviewed the routine MRI images of all patients, including breast composition defined by the amount of fibroglandular tissue (FGT) (fat, scattered, heterogeneous, or extreme), background parenchymal enhancement (BPE) (minimal, mild, moderate, and marked), size (longest diameter), shape (round or oval and irregular), margin (smooth, irregular, and star-shaped), internal enhancement pattern (homogeneous, heterogeneous, rim enhancement, and dark internal septations), time-signal intensity curve (TIC) (persistent, plateau, and washout), and lymph nodes enlargement (yes or no). Both physicians were blinded to the pathological results. Inconsistencies concerning morphological features were agreed upon by consensus.

ADC diagram can be automatically generated by DWI data of single exponential model through READ Y View software of GE AW4.7 workstation. The original image scanned by MAGiC sequence is post-processed by MAGiC software on GE host, and syMRI T2WI, T1 Mapping, T2 Mapping, and PD Mapping are automatically generated before and after enhancement. The tumor’s location is determined by referring to the enhanced DISCO sequence, and the ROI is synchronously copied to another atlas. The ROI delineation plane and size before and after enhancement are kept consistent. ADC values and T1, T2, and PD were respectively recorded. The ROI delineation methods included holistic sketch, i.e., ROI along the edge of each layer of the lesion, which was recorded as “tumor”; maximum plane delineation method, i.e., the second ROI was sketched by tumor outline method at the maximum level of the lesion, and was recorded as “stand”. In order to reduce the effect of partial volume effect, the ROI was slightly smaller than the edge of the lesion. Also, we tried to avoid the cystic degeneration, hemorrhage, and necrosis visible to the naked eye on the multi-phase enhanced DISCO images. The method for delineating the most obvious enhancement included a combination with DISCO images, where the third ROI was delineated in the most obvious enhancement of the lesions, marked as “local” (the range of ROI was 1.0-2.5 cm^2^ according to the size of the tumor). T ^+^ and P ^+^ represented relaxation time and proton density before and after enhancement, respectively. “ΔT%” and “ΔPD%” [ΔT%=(T-T^+^)/T, ΔPD%=(PD-PD^+^)/PD] represented the relative change rate in T values between pre- and post-contrast scanning.

### Statistical analysis

SPSS 26. 0 software (IBM company) and R software version 4.1.2 (http://www.R-project.org) were used for statistical analysis. The consistency between observers was evaluated by the intraclass correlation coefficient (ICC). The variables between benign and malignant groups were compared using the Mann-Whitney U test, Student’s t-test, Chi-square test, or Fisher’s exact test as appropriate. Multivariate Logistic regression was used to screen independent risk factors and establish a model. The combined nomogram incorporated various independent risk factors based on multivariate analysis. The area under ROC curve (area under the curve, AUC) was used to evaluate the discrimination of parameters and models, and the Delong test was used to compare the parameters and model AUC. The decision curve analysis (DCA) was used to evaluate the clinical utility of the model, while the calibration curve assessed the fitness of the model. P < 0.05 was considered to be statistically significant. Limited by the small sample size, the bootstrap resampling method (1,000 times) was used for internal verification of the model.

### Model establishment

#### Quantitative relaxation model

In order to determine which ROI delineation method has the best predictive efficacy, multivariable logistic regression analysis was used to screen out independent risk factors and establish a diagnostic model for the statistically significant parameters in the univariate analysis of the same ROI delineation method. The relaxation model of the best ROI delineation method was used in the final model.

#### BI-RADS model

The MRI signs of four types of BI-RADS masses were compared between benign and malignant groups, and the independent influencing factors with statistical significance were screened out by multivariate logistic regression analysis, based on which the prediction model of BI-RADS was established.

#### Joint diagnosis model

The quantitative parameters of SyMRI and DWI of the best ROI drawing method were combined with the DISCO enhanced MRI signs, and the nomogram was drawn.

## Results

A total of 98 patients with BI-RADS 4 types of breast mass lesions (all unilateral, all-female) were included for analysis. The patients were 22-78 years old, with an average age of 48.78 ± 11.24 years. There were 65 patients with malignant lesions and 33 with benign lesions.

### Quantitative relaxation model (model A)

The quantitative parameters of the three ROI measurement methods had good consistency (repeatability and reproducibility) within the same observer and among different observers (0.8313-0.9999). There were significant differences in T2, T2^+^, Δand T1%_local_ among benign and malignant groups (**Table 1**). Multivariate logistic regression analysis showed that T2_tumor_, T2_stand_, T2_local_, ΔT1%_tumor_, ΔT1%_stand_, and ΔT1%_local_ were independent factors affecting the malignancy of the lesions (**Table 2**). The relaxation parameters of the same ROI method were combined to form the diagnostic model, and the maximum slice drawing relaxation model (Model A: T2_stand_, ΔT1%_stand_) had the best differential diagnostic efficiency (AUC=0.887). Moreover, model A had higher diagnostic performance than that of strengthening the most obvious drawing relaxation model (AUC = 0.887 vs. 0.823, P= 0.0257), but similar to that of the overall drawing relaxation model (AUC = 0.887 vs. 0.850, P= 0.1864).

**Table 1:** Comparison of imaging characteristics between benign and malignant groups.

Variables	Malignant (n=68)	Benign (n=33)	*p*-value
T1_tumor_(ms)	1400.25 ± 321.78	1461.73 ± 573.28	0.498
T2_tumor_(ms)	81.00(77.42,87.84)	87.00(84.92,100.34)	<0.001
PD_tumor_(pu)	66.4(55.85,75.22)	65.50(60.85,81.98)	0.241
T1^+^_tumor_(ms)	574.5(536.25,626.50)	652.50(487.67, 1215.67)	0.051
T2^+^_tumor_(ms)	69.73 ± 6.98	76.54 ± 7.46	<0.001
PD^+^_tumor_(pu)	79.48 ± 16.20	81.37 ± 17.96	0.599
ΔT1%_tumor_(%)	57.94(53.71,64.91)	38.57(22.43,57.81)	<0.001
ΔT2%_tumor_(%)	14(11.33,17.25)	15.66(12.17,20.25)	0.073
ΔPD%_tumor_(%)	21.97 ± 11.93	25.53 ± 17.77	0.305
ADC_tumor_(×10-3 mm2/s)	1.04(0.92,1.15)	1.37(1.11,1.51)	<0.001
T1_stand_(ms)	1304(1150.42,1455.33)	1213.67(1102.58,1833.83)	0.827
T2_stand_(ms)	79(73.67,86.75)	88.5(86.17,101.00)	<0.001
PD_stand_(pu)	66.4(55.85,75.22)	65.5(60.85,81.98)	0.241
T1^+^_stand_(ms)	575.11(536.25,629.42)	652.5(487.67,1215.67)	0.058
T2^+^_stand_(ms)	64(59.84,69.00)	73(67.50,82.67)	<0.001
PD^+^_stand_(pu)	79.48 ± 16.20	81.37 ± 17.96	0.599
ΔT1%_stand_(%)	56.13(48.24,61.24)	43.18(35.80,52.51)	<0.001
ΔT2%_stand_(%)	17.85 ± 6.55	20.12 ± 7.97	0.136
ΔPD%_stand_(%)	21.97 ± 11.93	25.53 ± 17.77	0.305
ADC_stand_(×10^-3^ mm^2^/s)	1.03(0.91,1.14)	1.32(1.09,1.51)	<0.001
T1_local_(ms)	1362(1148.50, 1525.83)	1254(989.83,1871.33)	0.688
T2_local_(ms)	79.48 ± 10.30	90.31 ± 10.41	<0.001
PD_local_(pu)	64(55.18,75.85)	65.4(61.80,83.05)	0.153
T1^+^_local_(ms)	575(529.00,649.00)	602.33(478.00,1092.67)	0.100
T2^+^_local_(ms)	64(59.83,69.00)	73(63.83,82.67)	<0.001
PD^+^_local_(pu)	75.8(67.75,86.70)	79(66.80,94.63)	0.417
ΔT1%_local_(%)	56.78(49.34,61.65)	39.51(19.78,55.83)	<0.001
ΔT2%_local_(%)	17.53 ± 7.65	17.7 ± 9.23	0.920
ΔPD%_local_(%)	22.46 ± 13.77	27.03 ± 18.26	0.169
ADC_local_(×10^-3^ mm^2^/s)	0.99(0.87,1.12)	1.39(1.14,1.56)	<0.001
Size(mm)	18.42(13.40,23.38)	16.23(10.47,29.56)	0.749
Shape			0.017
Round or oval	19(29.2)	18(54.5)	
Irregular	46(70.8)	15(45.5)	
Margin			0.006
Smooth	4(6.2)	9(27.3)	
Irregular	48(89.2)	22(66.7)	
Star-shaped	13(20)	2(6.1)	
Internal Enhancement Pattern			<0.001
Homogeneous	2(3.1)	10(30.3)	
Heterogeneous	49(75.4)	16(48.5)	
Rim enhancement	12(18.5)	3(9.1)	
Dark internal septations	2(3.1)	4(12.1)	
TIC			<0.001
persistent	1(1.5)	9(27.3)	
plateau	23(35.4)	16(48.5)	
washout	41(63.1)	8(24.2)	
FGT			0.869
Fat	3(4.6)	2(6.1)	
Scattered fifibroglandular tissue	4(6.2)	1(3.0)	
Heterogeneous fifibroglandular tissue	52(80.0)	28(84.8)	
Extreme fifibroglandular tissue	6(9.2)	2(6.1)	
BPE			0.251
Minimal	1(3.0)	1(1.5)	
Mild	16(48.5)	42(64.6)	
Moderate	14(42.4)	16(24.6)	
Marked	2(6.1)	6(9.2)	
lymph nodes enlargement			0.079
no	52	21	
yes	13	12	

ADC, apparent diffusion coefficient; T1, transverse relaxation time; T2, longitudinal relaxation time; PD, proton density; T ^+^ and PD^+^, the quantitative values after enhancement.

**Table 2:** Parameters associated with breast cancer diagnosis in multivariable logistic regression analysis.

Variables	*p value*	OR	95%CI
T2_tumor_	0.014 ^*^	0.867	0.774-0.972
T2^+^_tumor_	0.580	1.038	0.909-1.187
ΔT1%_tumor_	<0.001^*^	1.070	1.031-1.110
T2_stand_	0.001^*^	0.761	0.651-0.891
T2^+^_stand_	0.445	1.039	0.942-1.146
ΔT1%_stand_	0.011^*^	1.078	1.018-1.142
T2_local_	0.039^*^	0.926	0.860-0.996
T2^+^_local_	0.403	0.969	0.899-1.044
ΔT1%_local_	0.001^*^	1.073	1.031-1.117
ADC_tumor_	<0.001^*^	0.003	0.000-0.038
ADC_stand_	0.001^*^	0.048	0.008-0.299
ADC_local_	<0.001^*^	0.002	0.000-0.025
Shape	0.961	0.971	0.302-3.129
margin	0.045^*^	3.241	0.302-3.129
internal enhancement pattern	0.695	1.162	0.548-2.465
TIC	<0.001^*^	4.594	2.013-10.483

OR, odds ratio; CI, confidence interval; TIC, time–signal intensity curve; ADC, apparent diffusion coefficient; ^*^ p< 0.05.

### BI-RADS model (model B)

**Figure 1:**
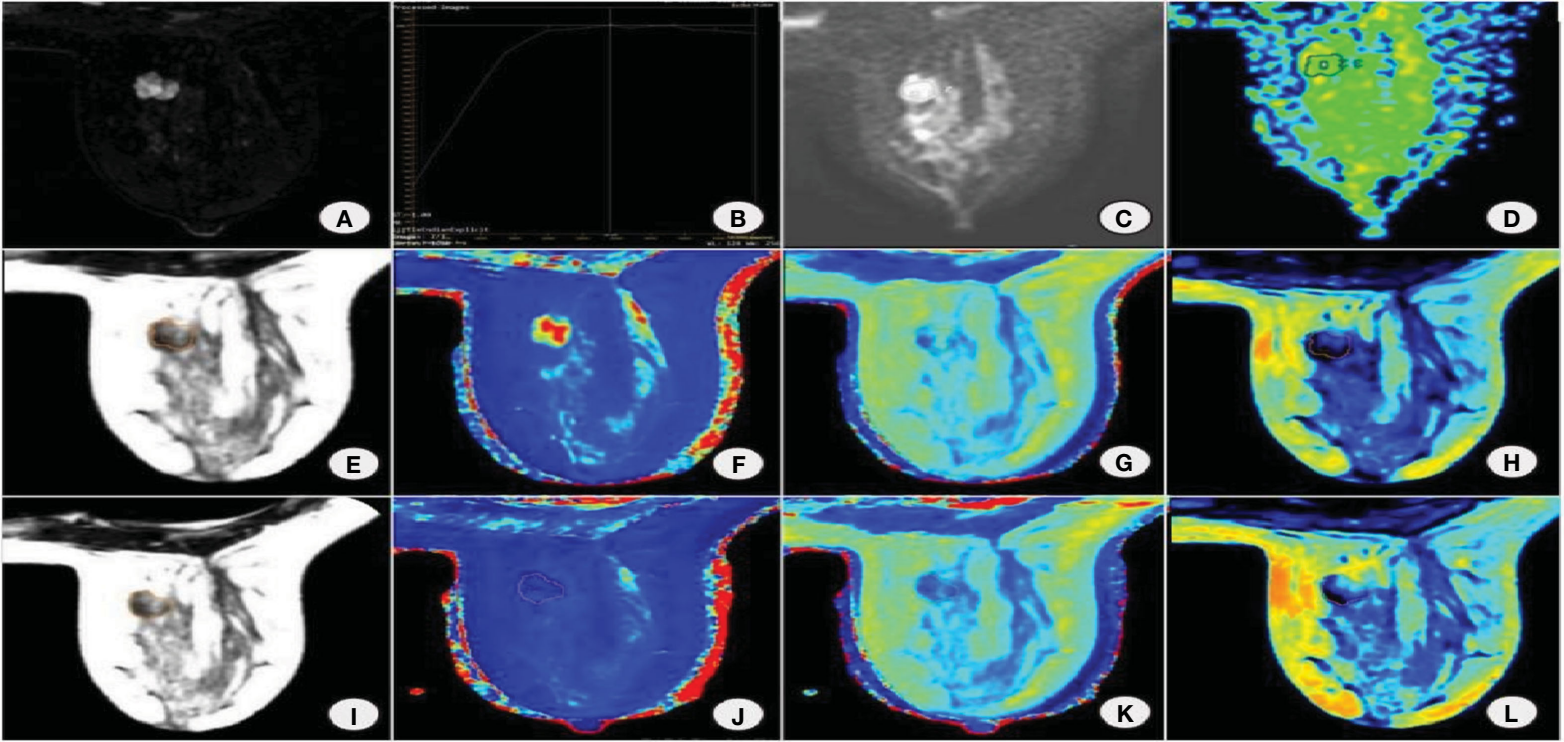
Female, 38 years old with right breast fibroadenoma. **(A)** DISCO enhancement image showing an irregular mass with irregular margin and uneven enhancement in the right breast inner quadrant. **(B)** TIC is a plateau. **(C)** DWI shows mass diffusion limitation. **(D)** ADC pseudocolor map. Image reconstruction with MAGiC. **(E)** SyMRI T2WI. **(F)** T1 map. **(G)** T2 map. **(H)** PD map. **(I)** SyMRI T2WI enhancement. **(J)** T1 enhancement map. **(K)** T2 enhancement map. **(L)** PD enhancement map.

**Figure 2:**
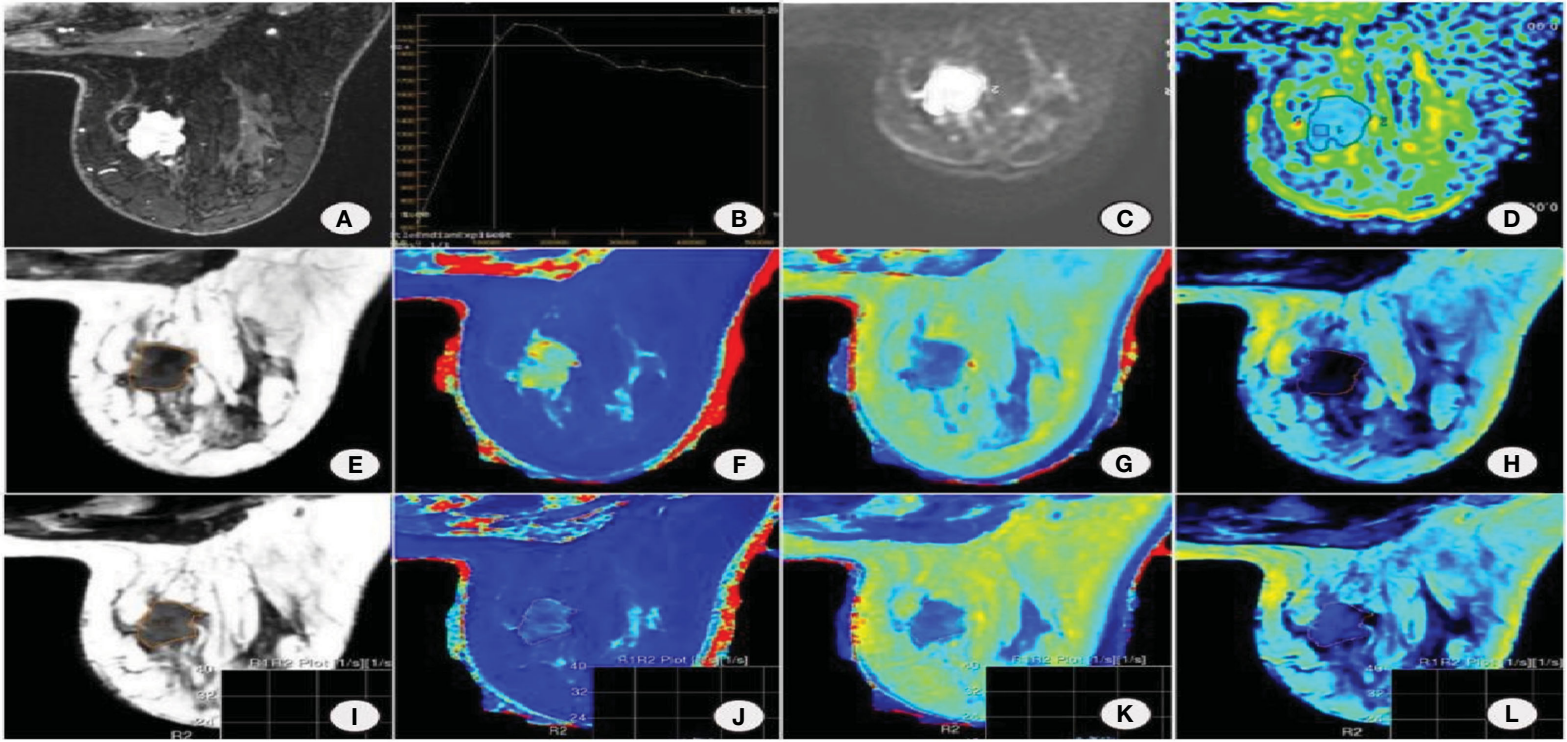
Female, 53 years old with non-special invasive carcinoma of the right breast. **(A)** DISCO enhancement image showing irregular mass with irregular margin and uneven enhancement in the upper inner quadrant of the right breast. **(B)** TIC is a washout. **(C)** DWI shows mass diffusion limitation. **(D)** ADC pseudocolor map. Image reconstruction with MAGiC. **(E)** SyMRI T2WI. **(F)** T1 map. **(G)** T2 map. **(H)** PD map. **(I)** SyMRI T2WI enhancement. **(J)** T1 enhancement map. **(K)** T2 enhancement map. **(L)** PD enhancement map.

Significant differences were found in morphology, margin, internal enhancement, and TIC curve between benign and malignant groups. Edge and TIC curves were independent factors affecting the malignancy of the lesion. BI-RADS model was constructed by two MRI features of edge and TIC curve (**Figures 1A-L**, **2A-L**).

### Joint diagnostic model (model C)

There were significant differences in ADC_tumor_, ADC_stand,_ and ADC_local_ between benign and malignant groups, and their AUC values were 0.816, 0.811, and 0.861, respectively. There was significant difference in AUC between ADC_local_, ADC_tumor_ and ADC_stand_ (Z = 1.980, 1.966 P =0.0478, 0.0493 respectively). Therefore, ADC_local_ was incorporated into the final joint diagnostic model.

**Figure 3:**
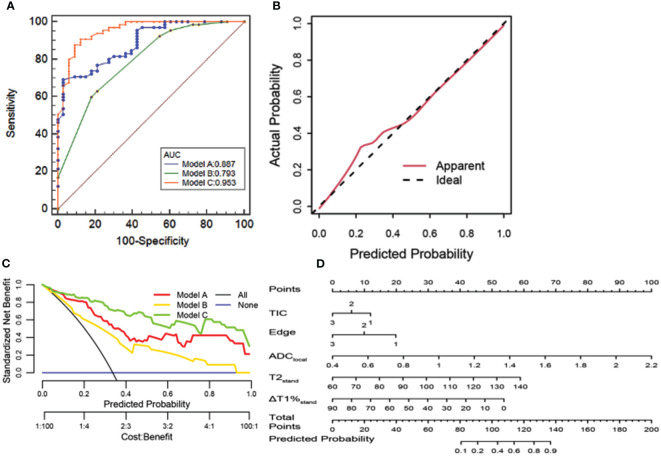
**(A)** ROC curve analysis. Model C has the best diagnostic performance (AUC = 0.953), followed by models A and B (AUC = 0.887, 0.793, and 0.856, all p < 0.05). **(B)** Calibration curve analysis. The closer the red line is to the dotted line, the better the model’s prediction ability. **(C)** The clinical decision curve of the predictive model. **(D)** The developed radiomics nomogram for predicting benign and malignant BI-RADS 4 lesions.

A joint diagnostic model (model C) was established using the quantitative parameters of SyMRI and DWI of the best ROC method combined with DISCO to enhance MRI features. The ROC curve results showed that model C had the best differential diagnosis efficiency compared with model A and model B (AUC=0.953) (**Table 3**, **Figures 3A, B**), and the AUC differences were statistically significant (Z= 2.794, 3.658, *P* all<0.01). There was no significant difference in diagnostic efficiency between model A and model B (Z=1.680, *P*=0.0930). When the threshold probability was 10% and 100%, the net benefit of the combined model (model C) was higher than that of other single parameter models. DCA showed that the net benefit of model 3 was better than that of the other models between threshold probabilities of 10%–100% (**Figure 3B**). The calibration curve of model C showed that the prediction probability of the model had good agreement with the actual probability (**Figure 3C**, P=0.762), and the sensitivity, specificity, positive predictive value (PPV), negative predictive value (NPV), and the accuracy of model 3 were 87.88%, 90.77%, 93.64%, and 82.88%, respectively.

**Table 3:** Diagnostic performance of different prediction models in differentiating BI-RADS four types of benign and malignant breast lesions.

Model	Variable	AUC	95% CI	Spe. (%)	Sen. (%)	PPV (%)	NPV (%)
Model 1	T2_stand_, ΔT1%_stand_	0.887	0.509, 0.769	69.23	96.97	97.82	61.57
Model 2	edge, TIC	0.793	0.699, 0.887	63.08	78.79	85.40	52.03
Model 3	edge, TIC, ADC_local_, T2_stand_,ΔT1%_stand_	0.953	0.593, 0.985	90.77	87.88	93.64	82.88

Sen., sensitivity; Spe., specificity; PPV, positive predictive value; NPV, negative predictive value.

## Discussion

In the present study, we found that using the maximum slice delineation method to measure MAGiC quantitative parameters and ADC value to measure ADC value at the most obvious enhancement was the best approach for the diagnosis of BI-RADS four types of mass lesions. Furthermore, the diagnostic model of SyMRI and DWI quantitative parameters combined with DISCO could further improve the diagnostic efficiency of BI-RADS 4 types of mass lesions, and the line diagram based on this model could be used as an auxiliary forecasting tool.

In the past, the clinical application of MR relaxation quantitative technique was limited by scanning time or high background noise, as the quantitative research on breast relaxation was mainly focused on T2 and T2 * relaxation time (17). In their study, Seo et al. showed that the T2* value of invasive carcinoma was higher than that of breast ductal carcinoma in situ, and higher histological grade was associated with higher signal intensity on T2WI and longer T2* relaxation time. That study was limited to the differential diagnosis of invasive ductal carcinoma and intraductal carcinoma in situ, while in this study, we comprehensively analyzed the feasibility of a variety of quantitative techniques in the differential diagnosis of different types of benign and malignant breast lesions (18). Multivariate Logistic regression analysis showed that T2_tumor_, T2_stand,_ and T2_local_ are independent factors affecting the malignancy of four types of BI-RADS breast lesions. Compared with the T2 value (82.69 ms) measured by Liu et al. by using the most obvious enhancement method on 1.5T MRI, the T2 _local_ value in the present study was lower (79.48ms) (19). Moreover, Jung et al. used a 3.0T SyMRI sequence to image, and the T2_tumor_ (84.75 ms) measured by the global sketching method was higher than that of the T2_tumor_ (81.00ms) in this study (20), which might be because the breast cancers included in this study had more complex heterogeneity. Although the scanning equipment and the types of cases included were different, studies confirmed that the T2 value of malignant lesions was significantly lower than that of benign lesions, which is because the relaxation time is mainly affected by tissue water content. The greater the free water content, the longer the T2 relaxation time (21). The proliferation rate of cancer cells in malignant breast lesions is fast, and necrotic substances and lymphocytes/plasma cells infiltrate the intercellular space, decreasing extracellular space and free water content. The cell density of benign breast lesions is relatively small, and the extracellular stroma is lower than that of malignant lesions. There is more tissue-free water, so the T2 relaxation time of malignant breast lesions is shorter than that of benign breast lesions. At the same time, in benign lesions, such as myxoid degeneration of fibroadenoma and interstitial edema caused by chronic inflammation of adenosis, T2 relaxation time was prolonged.

In our study, we not only measured the focal relaxation time before and after the contrast injection, but also calculated the relative rate of change of the relaxation time (ΔT%), and the results showed that the quantitative values of breast lesions changed differently after enhancement, ΔT1%tumor, ΔT1%stand, and ΔT1%local were independent factors affecting the malignancy of the BI-RADS 4 lesions. Therefore, post enhanced MAGiC scanning is of great value for the differentiation of benign and malignant tumors. Which were related to the characteristics of contrast medium and the microvascular environment of tumor tissue. When the contrast agent was intravenously injected, the malignant lesions with strong metabolism and a large number of immature neovascularization are clearly seen, while the basement membrane of neovascularization is incomplete or missing, resulting in increased vascular permeability and blood flow (22, 23). Contrast agents could quickly enter the tumor micro vessels and be quickly cleared while the T1 value of the lesions was gradually restored. Also, as the contrast media of benign lesions was still concentrated in the lesions, ΔT1%_tumor_, ΔT1%_stand_, and ΔT1%_local_ of malignant lesions were larger than those of benign lesions.

In the present study, we combined the relaxation parameters of the same ROI method to form a diagnostic model: MAGiC_tumor_ (T2_tumor_, ΔT1%_tumor_); MAGiC_stand_ (T2_stand_、ΔT1%_stand_); MAGiC_local_ (T2_local_, ΔT1%_local_). The results of ROC curve showed that MAGiC_stand_ was the most effective parameter in the differential diagnosis of benign and malignant breast masses of BI-RADS (AUC=0.887). Meng et al. explored the feasibility and diagnostic efficacy of SyMRI in differentiating nasopharyngeal carcinoma from benign nasopharyngeal hyperplasia, finding that the T2 value obtained from the largest plane of the lesion had the highest diagnostic accuracy, which was similar to the results of the present study (24). There was a significant difference in AUC between MAGiC_stand_ and MAGiC_local_ (Z=1.966, *P*=0.0257). The reason for the analysis is that the most obvious enhancement part of the tumor reflects the area with the densest cells and less free water content in the tumor tissue; however, because of the heterogeneity within the tumor, the most obvious enhancement site in the tumor cannot reflect the relaxation of the lesion as a whole. On the other hand, the method for delineation of most obvious regions through enhancement is easily affected by the subjective factors of the observer, resulting in different positions of ROI for different observers, and there are also differences in the measured values. There was no significant difference in AUC between MAGiC_stand_ and MAGiC_tumor_ (Z=1.321, *P*= 0.1864). The analysis is performed due to the reduction in differences caused by tumor heterogeneity, as the necrotic and cystic areas are avoided when drawing ROI. In addition, the ROI is slightly smaller than the edge of the focus, and the interference caused by the partial volume effect on the measurement can be eliminated as much as possible. However, the whole product drawing method is time-consuming and requires a heavy workload. In routine application, choosing the tumor maximum section method to measure SyMRI quantitative parameters is recommended, as this approach is simple to operate and has high repeatability.

The present study showed that the two factors of focus edge and TIC curve were independent influencing factors for the diagnosis of malignancy of BI-RADS 4 types of breast masses, which may be because malignant tumor cells have different growth rates, pull the surrounding tissue, and make their edges irregular. Malignant tumor cells secrete angiogenic factors to promote the formation of capillaries in the process of growth, their permeability is high, and contrast medium outflow is faster. Most of the TIC curves are platform type or outflow type. Model B (BI-RADS) was established by the combination of the above parameters, and the AUC for distinguishing benign and malignant BI-RADS 4 types of mass lesions was 0.749, suggesting that DISCO enhancement had certain diagnostic effectiveness for benign and malignant breast lesions, but it should be combined with multiple quantitative parameters to improve the efficiency of differential diagnosis.

It was found that the levels of ADC_tumor_, ADC_stand,_ and ADC_local_ in BI-RADS 4 types of malignant breast mass lesions were significantly lower than those in benign lesions (*P* all < 0 05). According to the analysis of the reasons, the tissue cells in the malignant lesions grew vigorously and proliferated rapidly. At the same time, the cell density and the ratio of nucleus to cytoplasm increased, which led to the limitation of the diffusion of water molecules. In this study, ROC curve analysis showed that the ADC_local_ value had the best value in the differential diagnosis of 4 types of breast mass lesions in BI-RADS (AUC=0.861). In this study, the difference in AUC between ADC_local_, ADC_tumor,_ and ADC_stand_ was statistically significant, which may be because ADC_local_ represents the area with the most obvious tumor enhancement, the densest cells, and the most limited dispersion of free water molecules. Some studies have also found a good correlation between the ADC value of the low-value area and the dense area of tumor tissue (25). At the same time, the distribution of local tumor cells was denser, and the heterogeneity of the tumor was relatively small. Although DWI can make up for the low diagnostic specificity of MRI to some extent, due to the influence of the b value and field strength, there are great differences in diagnostic sensitivity and specificity of ADC values reported in previous studies (26). Therefore, it is not sufficient to only rely on the DWI ADC value for differential diagnosis in a clinical setting; conventional scan sequence MRI features and a variety of quantitative parameters also provide information that should not be ignored.

The results of this study showed that SyMRI combined with DWI and DISCO multi-parameter combination model could further improve the differential diagnosis efficiency of BI-RADS four types of breast mass lesions, which is higher than that of BI-RADS and MAGiC model alone, with an AUC of 0.953, sensitivity of 90.77%, specificity of 87.88%, the positive predictive value of 93.64%, and negative predictive value of 82.88%. When the prediction probability was 10% to 100%, the net benefit of model C was better than that of other single technical models. Therefore, the combination of multi-parameters can make full use of the advantages of various technologies to reflect more comprehensive tumor information. The nomogram can obtain the probability of breast cancer by simple addition, for the patients with BI-RADS 4 benign lesions, as the quantitative parameters of T1%stand, T2stand, ADClocal value increase, their score rises, and the total score increases. Suggesting their risk probability of malignant lesions also increase, then for such patients, they will get more clinical attention, the follow-up period should be shortened, or suggest patients further examination for differential diagnosis and so on, which helps guide physicians to formulate individualized treatment plans for patients. Finally, the calibration curve was used to verify the calibration degree of the multi-parameter model. The results showed that the prediction probability of the model fitted well with the actual probability and had a good predictive ability, which suggested it was a simple and non-invasive auxiliary diagnosis method for the diagnosis of breast cancer.

The present study has the following limitations: (1) in this study, SyMRI was combined with DWI and DISCO parameters to establish a nomogram, revealing good prediction results for the differentiation of benign and malignant BI-RADS 4 types of lesions. Nonetheless, due to the sample size, we did not perform external cohort validation of the model. However, our study continues, and the focus of subsequent work will be on collecting more sample sizes for further validation of the model. (2) This study only included the lesions with mass enhancement shown by DISCO images. Because the non-mass enhancement lesions were mixed with normal glandular tissue, it was difficult to draw the region of interest. Also, as the number of cases was smaller, it was not included in the study for analysis. Many ductal carcinomas *in situ* showed non-mass enhancement, which will continue to supplement the value of SyMRI in the differential diagnosis of benign and malignant non-mass-enhanced lesions. (3) This was a single-center study with a small sample size and a lower number of BI-RADS 4 benign tumors vs. malignant tumors, which may lead to potential statistical deviation, so it is necessary to expand the sample size for the multicenter study.

To sum up, SyMRI combined with DWI and DISCO multi-parameter diagnosis model could further improve the differential diagnostic value of BI-RADS 4 types of benign and malignant mass lesions. Also, the line diagram based on this model has the advantages of visualization and interpretability, can help confirm the diagnosis, develop a more rational treatment plan, and possibly avoid unnecessary biopsies, especially for BI-RADS category 4 lesions. which is expected to provide an objective basis for the individualized treatment of patients with breast cancer.

## Data availability statement

The original contributions presented in the study are included in the article/**Supplementary Material**. Further inquiries can be directed to the corresponding author.

## Ethics statement

The studies involving humans were approved by Ethical Clearance Certificate of Ningxia Medical University General Hospital Scientific Research Ethics Committee. The studies were conducted in accordance with the local legislation and institutional requirements. The participants provided their written informed consent to participate in this study. Written informed consent was obtained from the individual(s) for the publication of any potentially identifiable images or data included in this article.

## Author contributions

HH: Conceptualization and writing. MS: Conceptualization. ZT: Formal analysis NG: Data collection. JM: Data collection. ZW: Supervision.

## Supplementary material

The Supplementary Material for this article can be found onlineat: https://www.frontiersin.org/articles/10.3389/fonc.2023.1180131/full#supplementary-material
