# Supplementary material for: Multiparametric MRI model with synthetic MRI, DWI multi-quantitative parameters, and differential sub-sampling with cartesian ordering enables BI-RADS 4 lesions diagnosis with high accuracy
Source: Front Oncol. 2024 Jan 5;13:1180131. doi: 10.3389/fonc.2023.1180131 (PMC10797086; doi:10.3389/fonc.2023.1180131)
Supplement: Supplementary file 1 [file Table_1.docx]

**Table S1.** Scan parameters for all sequences

| Parameters | T1WI | T2WI | DWI | Dyn DISCO+C | MAGiC |
| --- | --- | --- | --- | --- | --- |
| Sequence | FSE | STIR | Single-shot echo | Vibrant-Flex | MDME-FSE |
| Orientation | Ax | Ax | Ax | Ax | Ax |
| Fat suppression | No | Yes | Yes | Yes | No |
| TR (ms) | 613 | 4211 | 2976 | 3.7 | 4378 |
| TE (ms) | 7.5~37.3 | 85 | 72.6~166 | 1.3~21 | 19 |
| Slice thickness (mm) | 5 | 5 | 5 | 5 | 5 |
| Layer interval(mm) | 0.5 | 0.5 | 0.5 | 0 | 0.5 |
| FOV (mm) | 320×320 | 320×320 | 320×320 | 320×320 | 320×320 |
| Matrix | 384×300 | 320×288 | 128×140 | 320×320 | 320×256 |
| Acceleration factor | 2 | 2 | 2 | 2 | 2 |

FSE, fast spin-echo; STIR, short TI inversion recovery; Vibrant-Flex, Multi-contrast fast recovery of fast spin-echo; MDME,multiple-delay multiple-echo; FOV, field of vision.

**Table S2.** Included population pathology type

| Malignant lesions | | Benign lesions | |
| --- | --- | --- | --- |
| Pathology type | Number | Pathology type | Number |
| Ductal carcinomas in situ | 11 | Fibroadenomas | 20 |
| Invasive ductal carcinomas | 47 | Adenosis | 3 |
| Papillary carcinomas | 3 | Inflammation | 4 |
| Mucinous carcinoma | 1 | Benign papilloma | 5 |
| Invasive lobular carcinomas | 1 | Multiple retention cysts with chronic inflammation | 1 |

**Table S3.** ICC of three different ROI delineation methods in the same observer

| parameter | the overall drawing method | the maximum level drawing method | the strengthening most obvious drawing method |
| --- | --- | --- | --- |
| ADC | 0.9439 | 0.8889 | 0.9631 |
| T1 | 0.9914 | 0.9993 | 0.9992 |
| T2 | 0.9153 | 0.9617 | 0.9238 |
| PD | 0.95 | 0.9986 | 0.9964 |
| T1+ | 0.9999 | 0.9964 | 0.993 |
| T2+ | 0.8848 | 0.8313 | 0.8395 |
| PD+ | 0.9973 | 0.9909 | 0.9906 |

ICC,intraclass correlation coeffificient; ADC, apparent diffusion coefficient; T1, transverse relaxation time; T2, longitudinal relaxation time; PD, proton density; T ^+^ and PD^+^, the quantitative values after enhancement.

**Table S4.** ICC of three different ROI delineation methods in the different observer

| parameter | the overall drawing method | the maximum level drawing method | the strengthening most obvious drawing method |
| --- | --- | --- | --- |
| ADC | 0.9862 | 0.9465 | 0.9595 |
| T1 | 0.9979 | 0.9997 | 0.9986 |
| T2 | 0.9763 | 0.984 | 0.9646 |
| PD | 0.9916 | 0.9962 | 0.9989 |
| T1^+^ | 0.9932 | 0.9979 | 0.9995 |
| T2^+^ | 0.9745 | 0.9742 | 0.9687 |
| PD^+^ | 0.9854 | 0.9739 | 0.9952 |

**Table S5.** Comparison of the efficacy of the quantitative relaxation models of three ROI delineation methods in diagnosing BI-RADS 4 types of mass lesions

| Group | Standard error | 95%CI | *Z*-value | *P*-value |
| --- | --- | --- | --- | --- |
| MAGiCtumor VS MAGiCstand | 0.2610 | -0.0167~0.0857 | 1.321 | 0.1864 |
| MAGiCtumor VS MAGiClocal | 0.0301 | -0.0293~0.0886 | 0.984 | 0.325 |
| MAGiCstand VS MAGiClocal | 0.0287 | 0.00777~0.120 | 1.966 | 0.0257 |

MAGiC_tumor_, global outline relaxation model (T2_tumor_、∆T1%_tumor_); MAGiC_stand_, maximum slice delineation relaxation model（T2_stand_、∆T1%_stand_）; MAGiC_local_, outline relaxation model at the most obvious strengthening point（T2_local_、∆T1%_local_）.

**Table S6.** ROC curve analysis of ADC values of three ROI delineation methods

| parameter | AUC （95%CI） | Best threshold （× 10-3mm2/s） | Spe. (%) | Sen. (%) | PPV (%) | NPV (%) |
| --- | --- | --- | --- | --- | --- | --- |
|  |  |  |  |  |  |  |
|  |  |  |  |  |  |  |
| ADC_tumor_ | 0.816 （0.725，0.887） | 1.27 | 90.77 | 66.67 | 84.27 | 78.59 |
|  |  |  |  |  |  |  |
| ADC_stand_ | 0.811 （0.720，0.883） | 1.27 | 90.77 | 60.61 | 81.93 | 76.95 |
|  |  |  |  |  |  |  |
| ADC_local_ | 0.861 （0.776，0.922） | 1.07 | 69.23 | 87.88 | 91.83 | 59.21 |
|  |  |  |  |  |  |  |

Sen., sensitivity; Spe., specifificity; PPV, positive predictive value; NPV, negative predictive value.

**Table S7.** AUC comparison of three ROI delineation methods for ADC values of BI-RADS 4 types of breast lesions

| Group | Standard error | 95%CI | *Z-*value | *P-*value |
| --- | --- | --- | --- | --- |
| ADC_tumor_ VS ADC_stand_ | 0.0194 | 0.0332~0.0430 | 0.252 | 0.8011 |
| ADC_tumor_ VS ADC_local_ | 0.0225 | 0.0004~0.0886 | 1.98 | 0.0478 |
| ADC_stand_ VS ADC_local_ | 0.0251 | 0.0001~0.0987 | 1.966 | 0.0493 |

**Table S8.** AUC comparison of BI-RADS 4 types of breast lesions combined diagnosis model

| Group | Standard error | 95%CI | *Z-*value | *P-*value |
| --- | --- | --- | --- | --- |
| model A VS model B | 0.0561 | -0.0157~0.204 | 1.680 | 0.0930 |
| model A VS model C | 0.0234 | 0.0196~0.111 | 2.794 | 0.0052 |
| model B VS model C | 0.0436 | 0.0741~0.245 | 3.658 | 0.0003 |

model A, Quantitative relaxation model(T2_stand_，∆T1%_stand_); model B, BI-RADS model (edge, TIC); model C, Joint diagnosis model (edge,TIC,ADC_local_,T2_stand_,∆T1%_stand_).
